# *In vivo* ultrasound thermal ablation control using echo decorrelation imaging in rabbit liver and VX2 tumor

**DOI:** 10.1371/journal.pone.0226001

**Published:** 2019-12-05

**Authors:** Mohamed A. Abbass, Syed A. Ahmad, Neeraja Mahalingam, K. Sameer Krothapalli, Jack A. Masterson, Marepalli B. Rao, Peter G. Barthe, T. Douglas Mast

**Affiliations:** 1 Dept of Biomedical Engineering, University of Cincinnati, Cincinnati, Ohio, United States of America; 2 Dept of Surgery, University of Cincinnati, Cincinnati, Ohio, United States of America; 3 Dept of Environmental Health, University of Cincinnati, Cincinnati, Ohio, United States of America; 4 Guided Therapy Systems/Ardent Sound, Mesa, Arizona, United States of America; Northwestern University Feinberg School of Medicine, UNITED STATES

## Abstract

The utility of echo decorrelation imaging feedback for real-time control of *in vivo* ultrasound thermal ablation was assessed in rabbit liver with VX2 tumor. High-intensity focused ultrasound (HIFU) and unfocused (bulk) ablation were performed using 5 MHz linear image-ablate arrays. Treatments comprised up to nine lower-power sonications, followed by up to nine higher-power sonications, ceasing when the average cumulative echo decorrelation within a control region of interest exceeded a predefined threshold (− 2.3, log_10_-scaled echo decorrelation per millisecond, corresponding to 90% specificity for tumor ablation prediction in previous *in vivo* experiments). This threshold was exceeded in all cases for both HIFU (*N* = 12) and bulk (*N* = 8) ablation. Controlled HIFU trials achieved a significantly higher average ablation rate compared to comparable ablation trials without image-based control, reported previously. Both controlled HIFU and bulk ablation trials required significantly less treatment time than these previous uncontrolled trials. Prediction of local liver and VX2 tumor ablation using echo decorrelation was tested using receiver operator characteristic curve analysis, showing prediction capability statistically equivalent to uncontrolled trials. Compared to uncontrolled trials, controlled trials resulted in smaller thermal ablation regions and higher contrast between echo decorrelation in treated vs. untreated regions. These results indicate that control using echo decorrelation imaging may reduce treatment duration and increase treatment reliability for *in vivo* thermal ablation.

## Introduction

Hepatocellular carcinoma (HCC) is the most common primary malignant tumor in liver, especially in patients with cirrhosis (70–90% of all cirrhosis patients) [1, 2]. The liver is also one of the most common sites for secondary tumors, e.g. colorectal cancer liver metastases (CLM). Prevalence of CLM is affected by the incidence of colorectal cancer, the third most common cancer worldwide, since approximately 50% of patients with colorectal cancer develop CLM [3]. Treatment and prognosis of HCC depend on the tumor stage and status of the residual liver function [4].

Liver transplantation is considered the gold standard for HCC treatment for eligible patients (e.g., solitary tumor with normal portal pressure), but is limited by the availability of liver donors and by cost [1]. Another favorable therapeutic option for early-stage HCC patients with non-cirrhotic liver is hepatic resection. However, the overall resectability rate is low (20–30%) due to a combination of underlying chronic liver disease, tumor location (e.g., close to vascular structures or the diaphragm), and the multifocal nature of some HCC [5]. For CLM, surgical resection is the gold standard for treating isolated metastases in patients who are medically qualified for hepatectomy [6]. However, the number of CLM patients eligible for resection is < 20% due to unsuitable tumor locations and impaired residual liver function [7]. Thermal ablation (e.g., radiofrequency ablation [8], microwave ablation [9], laser interstitial thermal therapy [10], and ultrasound thermal ablation [11–13]) is the most appropriate option for HCC patients who are ineligible for transplantation or resection and with tumor size up to 3 cm, based on the Barcelona Clinic Liver Cancer classification system [14]. Thermal ablation may also provide a 5-year overall survival rate equivalent to surgical resection (47.6% vs. 56.0%) for patients with small CLM [15].

Monitoring and control of thermal ablation are essential to ensure treatment completion and to avoid complications due to overtreatment (e.g., hemorrhage, bowel injuries, and vascular thrombosis [16]). Magnetic resonance imaging [9, 17] (e.g., proton resonance frequency) provides a 3D temperature map of the treated region in near real time [18], but is limited by its cost [19] and need for magnetic resonance compatible equipment [20]. An attractive alternative method for monitoring thermal ablation is B-mode ultrasound imaging [21, 22], which is inexpensive, provides some real-time feedback on progression of thermal ablation, and avoids using ionizing radiation, unlike computed tomography (CT) [23]. However, formation of heat-induced gas bubbles obscures the visibility of tumor margins due to acoustic shadowing [24] which increases the probability of tumor recurrence.

To overcome the limitations of B-mode US imaging, other pulse-echo US imaging methods have been developed to guide thermal ablation. These methods have monitored thermal coagulation by tracking tissue stiffness and sound speed variations using cross-correlation between echo signals (e.g., elastography [25], harmonic motion imaging [26], acoustic radiation force, and echo strain imaging [22, 27]) or by quantifying changes in backscattered energy using M-mode [28], contrast enhanced US [29], real-time image fusion [30], and integrated backscatter imaging [31, 32]). These methods have some limitations associated with decorrelation between echo signals due to motion [33] or heat [34], as well as the presence of vapor clouds in the ablation region, potentially causing inaccurate prediction of thermal ablation [34–36].

In contrast, echo decorrelation imaging [35–37], a real-time pulse-echo US method, tracks decorrelation of echo signals over millisecond time scales to map thermal ablation effects by quantifying heat-induced decoherence of tissue reflectivity [38]. Echo decorrelation imaging has previously been demonstrated to accurately predict thermal ablation effects for multiple therapy modalities, including US ablation [28, 37, 39], radiofrequency ablation [35, 36, 40], and microwave ablation [41].

Echo decorrelation imaging has been successfully validated for controlling *ex vivo* US thermal ablation in bovine liver [42, 43] and in chicken breast [44]. Results from previous *ex vivo* studies indicate that preclinical translation of the proposed real-time control method is feasible with some considerations. These considerations include necessary modifications to stopping criteria previously employed in controlled *ex vivo* US experiments [42, 43], for consistency with *in vivo* tissue acoustical and anatomical characteristics. In addition, compensating the effect of artifactual echo decorrelation due to motion and noise is an essential consideration for controlling thermal ablation *in vivo*.

The ability of echo decorrelation imaging to monitor *in vivo* US treatments in rabbit liver and VX2 tumor was successfully demonstrated by Fosnight et al. [37], without real-time control. A motion and noise correction method [38] was used to correct cumulative echo decorrelation maps computed from stored echo data. Here, the same correction method was implemented to provide corrected cumulative echo decorrelation maps in real time, and was integrated with a real-time control algorithm previously validated for *ex vivo* ablation [42, 43].

The objective of this study was to investigate the feasibility of controlling *in vivo* US thermal ablation, including high-intensity focused ultrasound (HIFU) and unfocused (bulk) US ablation, using motion-corrected echo decorrelation imaging feedback in rabbit liver and VX2 tumor. Thermal ablation was assessed immediately after rabbit sacrifice by directly comparing treated tissue histology to corresponding echo decorrelation maps. Ablation outcomes and prediction capability of echo decorrelation imaging for controlled trials were compared with similar uncontrolled (i.e., not employing control by echo decorrelation imaging) *in vivo* US experiments previously reported by Fosnight et al. [37].

## Materials and methods

In this section, the experimental setup and procedures for controlled *in vivo* US thermal ablation in rabbit liver and VX2 tumor are described. More detailed description is provided in Ref. [45].

### Echo decorrelation imaging

Echo decorrelation imaging predicts local thermal ablation by quantifying heat-induced variations in backscattered echo signals over short time scales (e.g., milliseconds). Echo decorrelation maps were computed as

$$\Delta \left(y,z,t\right)=\frac{\beta^{2}\left(y,z,t\right)-{\left|R\left(y,z,t\right)\right|}^{2}}{\tau (\beta^{2}\left(y,z,t\right)+\bar{\beta^{2}\left(t\right)})/2}$$ (1)

In Eq 1, *y* and *z* are azimuth and range coordinates within an image frame, *R*(*y*, *z*, *t*) = 〈*I*(*y*, *z*, *t*)*I(*y*, *z*, *t* + *τ*)〉 is the position-dependent, zero-lag cross-correlation between sequential echo frames, computed by convolution of the conjugate product of complex echo frames, separated by an interframe time *τ*, with a spatial Gaussian window with width parameter *σ* = 1 mm. The integrated backscatter term *β*^2^(*y*, *z*, *t*) = 〈|*I*(*y*, *z*, *t*)|^2^〉〈|*I*(*y*, *z*, *t* + *τ*)|^2^〉 is computed by convolution of the magnitude-squared echo frames with the same Gaussian window and its spatial average is represented by $\bar{\beta^{2}\left(t\right)}$. Detailed derivation and analysis of echo decorrelation imaging is presented elsewhere [35, 36, 38, 42].

For each cycle, an ensemble-averaged echo decorrelation map was defined as

$$\bar{\Delta }\left(y,z,m\right)=\frac{1}{K-1}\sum_{k=1}^{K-1}\Delta (y,z,k\tau )$$ (2)

where *m* is the sonication cycle index, *y* and *z* are azimuthal and range coordinates, *K* is the number of frames recorded per cycle, and *k* is the frame index. Cumulative echo decorrelation maps were defined as the temporal maximum of the ensemble-averaged echo decorrelation map for each pixel position (*y*, *z*),

$$\Delta {\left(y,z,m\right)}_{\text{cum}}=\text{max}(\bar{\Delta }\left(y,z,m-1\right),\bar{\Delta }\left(y,z,m\right))$$ (3)

### Motion-corrected feedback control algorithm

The real-time feedback control algorithm employed here has been validated in *ex vivo* bovine liver experiments to control HIFU [42] and bulk US [43] ablation treatments. For better prediction and control performance, the motion and noise compensation method previously described by Hooi et al. [38] was integrated with the control algorithm, such that the effect of motion-induced decorrelation was corrected in real time. Corrected cumulative ensemble-averaged echo decorrelation maps (Δ_corr_) [37] for each therapy cycle were computed as

$$\Delta {\left(y,z,m\right)}_{\text{corr}}=\frac{\Delta {\left(y,z,m\right)}_{\text{cum}}-\Delta_{\text{sham}}}{1-\Delta_{\text{sham}}}$$ (4)

where Δ_sham_ is the cumulative ensemble-averaged echo decorrelation map computed for sham cycles (i.e., treatments with zero acoustic power). In the corrected decorrelation map, points where Δ(*y*, *z*, *m*)_corr_ < 0 were replaced by the minimum value of Δ_sham_.

The average of Δ(*y*, *z*, *m*)_corr_ < 0 inside the control ROI (Δ_avg_) was used as a feedback criterion to control HIFU or bulk US treatments. Therapy cycles were repeated until Δ_avg_ exceeded a prespecified control threshold (Δ_th_) or the sonication cycle index *m* exceeded the maximum number of therapy cycles (*M* = 18) [42].

**Fig 1:**
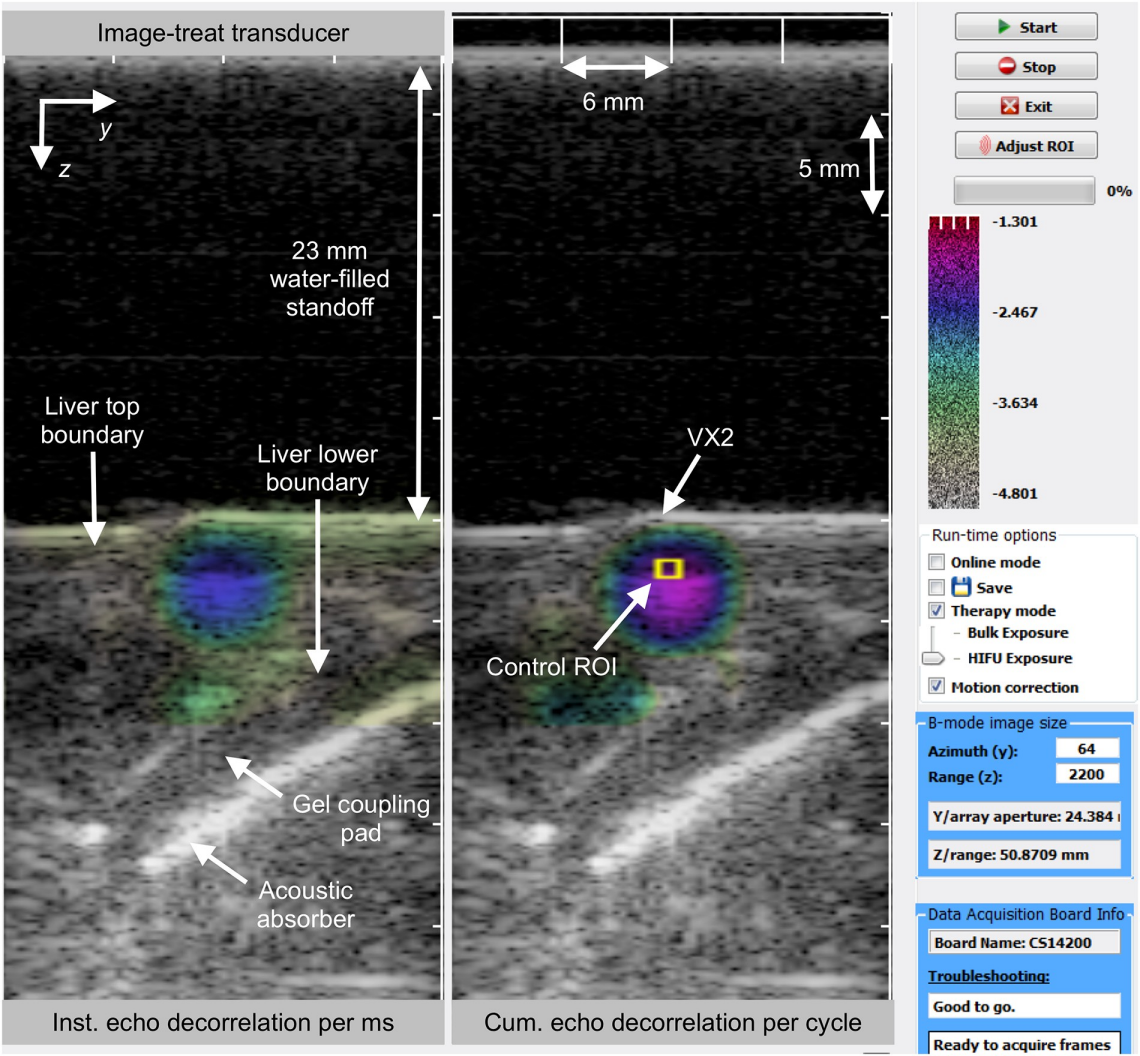
Graphical user interface of the C++ application used for *in vivo* US thermal ablation imaging and control. Left: instantaneous hybrid B-mode/echo decorrelation image. Right: cumulative echo decorrelation map for each therapy cycle, corrected in real time using decorrelation from sham ablation cycles; the control region of interest is bounded by a yellow line.

A graphical user interface (GUI) utilized in previous controlled *ex vivo* HIFU [42] and bulk US [43] thermal ablation experiments was modified by adding more features for better monitoring and control performance, as shown in Fig 1. The user selected the appropriate control ROI shape and size for HIFU or bulk US ablation. When needed, control and decorrelation ROIs were repositioned to fall within liver lobe boundaries.

### *In vivo* US ablation experiments

All animal procedures were performed according to a protocol approved by the University of Cincinnati (UC) Institutional Animal Care and Use Committee. New Zealand white rabbits were purchased by UC Laboratory Animal Medicine Services (LAMS) from Charles River Laboratories (Wilmington, MA). Rabbits were housed at LAMS under the care of skilled veterinary technicians and fed *ad libitum* except for overnight fasting (water allowed) immediately prior to surgery. VX2 tumor fragments were propagated by implantation in livers of carrier rabbits, starting from samples originating from Case Western Reserve University [46].

For tumor implantation, rabbits were sedated using ketamine (10 mg/kg) and xylazine (3 mg/kg) and anesthetized using isoflurane. VX2 tumor fragments were implanted in the three main liver lobes of 8 animals, each approximately 19 mm from the inferior liver lobe edge. Between tumor implantation and ablation procedures, rabbits were regularly assessed for pain based on standard physical manifestations (e.g., guarding, restlessness, lack of mobility, and abnormal postures) and analgesics (buprenorphine injection or fentanyl patch) were administered as required.

After two weeks tumor growth, US ablation experiments were performed on rabbit liver and VX2 tumor in open surgery. Ablation treatments and imaging were controlled by the Iris 2 US imaging and therapy system (Ardent Sound Mesa, AZ, USA) [47]. Custom dual mode, image-treat array transducers (64 element, 4.8 × 24.4 mm^2^ aperture, 5.35–5.50 MHz) performed pulse-echo US imaging (> 40% bandwidth, transmit focal depth 3.5 cm, *F*-number 4) and US ablation (maximum acoustic power 35 W). Transducers were integrated with a 23 mm standoff sealed by a transparent film (Tegaderm, 3M Health Care, St. Paul, MN) and filled with deionized, degassed water.

**Fig 2:**
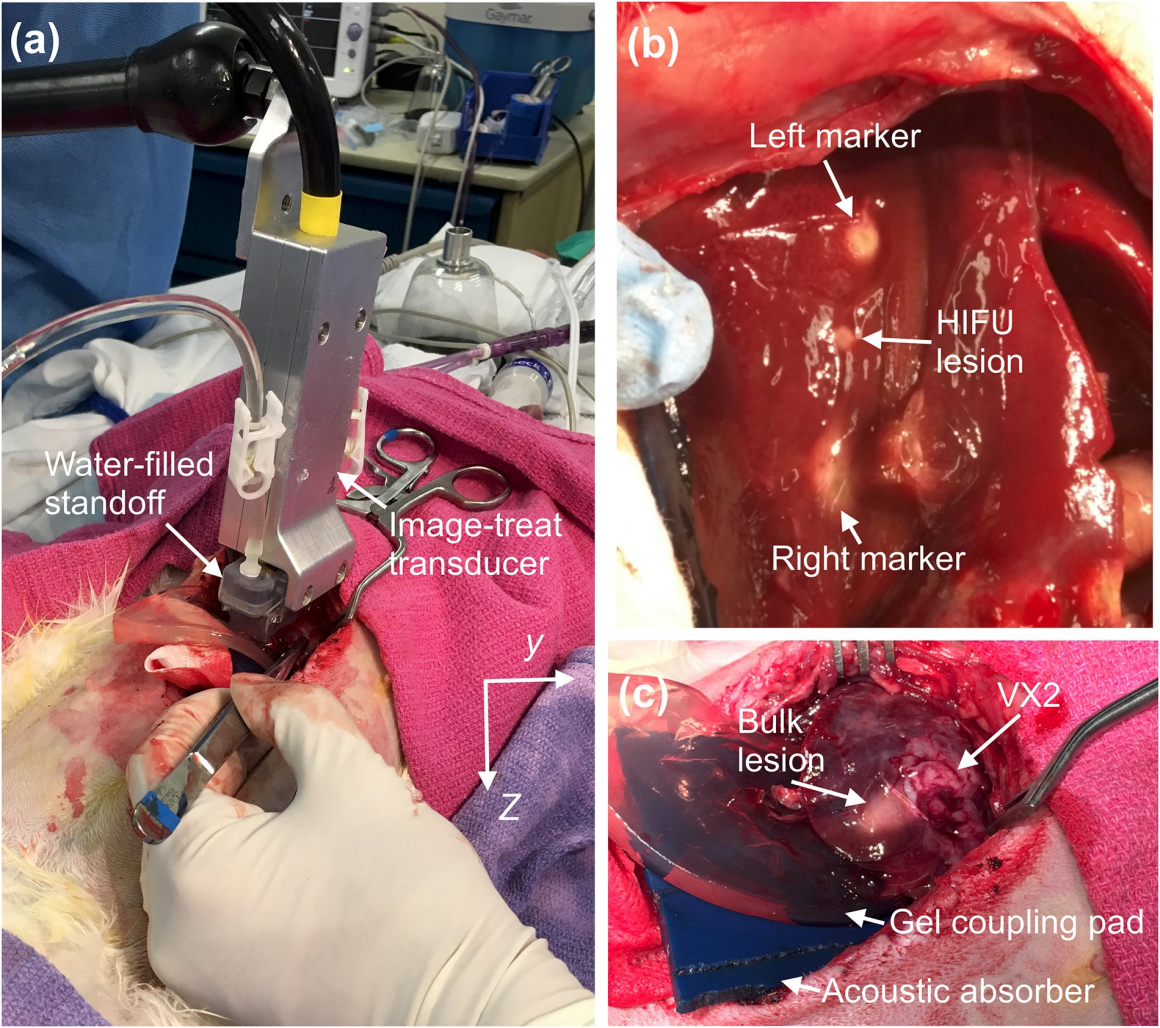
Experimental setup. (A) Image-treat array placed on the rabbit liver capsule during open surgery. (B) HIFU thermal ablation of liver followed by left and right marking exposures. (C) Bulk thermal ablation of VX2 tumor.

Before each treatment, the animal was sedated and its liver was exposed. Before starting US ablation, the tumor was located on each liver lobe surface by inspection and palpation. A standalone acrylic standoff with a footprint 21.0 mm in elevation and 38.0 mm in azimuth, identical to the standoff integrated with the US transducer, was used to center the transducer over the tumor surface by marking the standoff corners on the liver capsule with a skin marking pen (Accu-line Products Inc., Hyannis, MA). Thereafter, an acoustic gel pad (Aquaflex, Parker Laboratories Inc., Fairfield, NJ) and acoustic absorber (Precision Acoustics Ltd., Dorset, UK), each cut and thinned by hand, were placed beneath the target zone to minimize acoustic reflection and to constrain the shape of the liver lobe (Fig 2C). The array was aligned using a 3D positioning arm (NOGA Engineering Ltd., Israel) over the marked target zone (Fig 2A) and the tumor location was confirmed visually using the B-mode image on the Iris 2 system screen.

Ultrasound exposures were performed in cycles with sonication followed by pulse-echo imaging and RF data acquisition. During the imaging period, twenty beamformed RF echo frames were acquired with an 8.6 ms inter-frame time (frame rate 116 Hz). RF frames were sampled and digitized using a data acquisition card (14-bit, 33.3 MHz sampling rate; Compuscope 14200, Gage Applied). Digitized RF frames were processed by Hilbert transform to provide in-phase and quadrature (IQ) complex components, demodulated using a 5.0 MHz carrier frequency, and decimated by a factor of 6. Processed IQ frames were used to compute B-mode and echo decorrelation images according to Eqs 1–4 [42, 43].

At the end of US ablation experiments, the rabbit was sacrificed using Euthasol (200 mg/kg), and its liver was excised and placed in chilled 0.01M phosphate buffered saline solution. Within three hours after animal sacrifice, treated liver and VX2 tumor tissue were sectioned along the image plane and stained with 2% triphenyl tetrazolium chloride (TTC) vital stain. Treated tissue histology was assessed based on TTC stain uptake. For liver tissue, regions of full TTC uptake (stained red) were interpreted as untreated, and regions of partial or no TTC uptake (stained pale red or brown) were interpreted as treated [48]. For tumor tissue, regions of full TTC uptake (stained pale red) were interpreted as untreated and regions of partial or no TTC uptake (stained faint white or white) were interpreted as treated. For either tissue type, regions of no TTC uptake were considered fully ablated. TTC-stained sections were optically scanned at 1200 dpi (CanoScan 8800F, Canon, Tokyo, Japan). Of the two facing cross-sections, one was chosen for segmentation by examining sizes of the tumor and thermal ablation zone. The cross-section with the larger thermal ablation zone (or if those were equal, the larger tumor size) was chosen. Scanned histologic images were then manually segmented into untreated, treated, and fully ablated regions [37]. TTC-segmented sections were co-registered using a custom 2D rigid registration MATLAB application [42].

### Controlled HIFU ablation experiments

HIFU treatments were performed using the same timing sequence (0.7 s therapy and 2.2 s imaging per cycle) and control ROI (1 × 1 mm^2^) employed in previous controlled *ex vivo* HIFU experiments [42]. In each HIFU treatment, focused ultrasound was targeted to treat tissue at a single site, either within a VX2 tumor (*N* = 7) or within normal liver parenchyma (*N* = 7). The variable intensity sonication sequence previously tested in *ex vivo* bulk US ablation was employed. This sequence includes up to 9 cycles at a lower sonication intensity, followed by up to 9 cycles at higher intensity, with the goal of ensuring complete ablation treatment while avoiding overtreatment. The echo decorrelation threshold Δ_th_ was chosen as the optimal threshold for local ablation prediction in VX2 tumor computed by Fosnight et al. [37] in a similar *in vivo* study. This threshold was −2.3 (log_10_-scaled decorrelation per ms), which corresponded to 90% specificity and 43% sensitivity in the previous study [37].

For HIFU treatments (*N* = 14), the control ROI was placed 2 mm below the tissue surface. The variable sonication sequence began with 9 sham cycles, followed by 9 sonication cycles (5.35–5.50 MHz, 24% duty) with 24 W peak acoustic power (estimated spatial-peak, temporal-peak intensity *I*_SPTP_ = 1025 W/cm^2^) and up to 9 cycles with 28 W peak acoustic power (estimated *I*_SPTP_ = 1196 W/cm^2^), with treatments ending when Δ_avg_ within the control ROI exceeded Δ_th_ or when the sonication cycle index *m* exceeded the maximum number of therapy cycles *M*. The lower and higher acoustic power values employed here approximated the average and maximum values used by Fosnight et al. [37] for uncontrolled (i.e., not employing control by echo decorrelation imaging) *in vivo* HIFU experiments. Since these acoustic power levels were found in the previous *in vivo* study to consistently produce thermal ablation in rabbit liver with VX2 tumor [37], use of the same ranges here aimed to increase the likelihood of ablation completion. Controlled trials were compared with *in vivo* uncontrolled HIFU trials (*N* = 12) [37] (6–9 cycles, 5.0–5.4 MHz, 17.5–20.0% duty, 20–28 W peak acoustic power, 911–1351 W/cm^2^ estimated *I*_SPTP_).

After each HIFU treatment, two marking spots of thermal ablation were performed at the left and right of each HIFU ablation location, as shown in Fig 2B, to facilitate post-treatment tissue sectioning and for better registration of histologic and US images. Marking ablations were performed using controlled unfocused exposures fired from the first (1 to 10) or last 10 elements (55 to 64) of the transducer. For the marking exposures, a control ROI, with the same size as the control ROI for HIFU exposures, was positioned 2 mm below the tissue top surface and azimuthally at the center of each 10-element’s aperture (10.3 mm left or right from the focal point [37]). Marking exposures were controlled using the same control criteria employed for HIFU ablation. Unfocused sonications employed 6 s pulses (73.1% duty) with 53.8 W/cm^2^ *I*_SPTP_ up to a maximum of 9 therapy cycles, or ended when Δ_avg_ within the control ROI exceeded Δ_th_.

### Controlled bulk US ablation experiments

Bulk US treatments were performed using variable intensity sonication sequences using the same timing scheme (6.0 s therapy and 2.2 s imaging per cycle) as previous controlled *ex vivo* bulk US ablation experiments [43, 49]. For bulk US ablation, the entire array aperture was fired without electronic focusing, resulting in a heated zone of nominal width 24.4 mm. This treatment scheme provides heating rates and ablation volumes comparable to other bulk thermal ablation methods, such as RFA and MWA, and distinctly different from HIFU ablation.

Bulk US thermal treatments were controlled using the average-decorrelation criterion [43] with minor modifications in ROI shape to match the size of rabbit liver lobe cross-sections and the resulting shape of the thermal ablation zone. A *post hoc* analysis was performed on archived segmented tissue sections of treated, TTC-stained rabbit liver and VX2 tumor from previous *in vivo* bulk US ablation experiments (*N* = 10) [37] to compute the average and standard deviation of ablation zone widths, depths, and areas. Results of the *post hoc* analysis were compared with average ablation zone dimensions of the bulk *ex vivo* trials controlled using the average-decorrelation criterion [49]. Due to the smaller size of rabbit liver lobes, average ablation zone depths (11.2 ± 3.8 mm) and areas (2.4 ± 0.8 cm^2^) for rabbit liver were smaller than for bovine liver by approximately 30%. Hence, the control ROI depth used in previous *ex vivo* bulk experiments was reduced by 30% to 6 mm. For consistency, ROI area was kept the same by increasing the lateral distance between the rectangle edges by 50% on each side. The resulting control ROI was selected as 18 mm in width × 6 mm in depth.

For bulk US treatments (*N* = 10), the control ROI was placed 2 mm below the tissue surface. For some thinner liver lobes, the ROI was approximately centered between the top and bottom lobe boundaries. For controlled bulk US treatments, the variable sonication sequence began with 9 sham cycles, followed by 9 sonication cycles (5.35–5.50 MHz, 73.1% duty) with peak acoustic power 30 W (estimated *I*_SPTP_ = 48 W/cm^2^) and up to 9 cycles with peak acoustic power 35 W (estimated *I*_SPTP_ = 56 W/cm^2^), with treatments ending when Δ_avg_ within the ROI exceeded Δ_th_ or when *m* exceeded *M*. The lower and higher acoustic power values employed approximated the average and maximum acoustic powers used in the uncontrolled *in vivo* bulk US experiments [37]. Similar to the choice of acoustic power levels in the controlled HIFU experiments, matching of power levels with previous uncontrolled bulk US experiments aimed to enable complete ablation of the targeted treatment zones including VX2 tumor of diameter up to about 1.5 cm. Controlled trials were compared with these previous uncontrolled *in vivo* trials (*N* = 10, 7–9 therapy cycles, 5.0–5.4 MHz, 60–70.5% duty, 28–35 W peak acoustic power, 45–56 W/cm^2^ estimated *I*_SPTP_) [37].

### Data analysis

Tumor growth was assessed and effective tumor diameters were computed after tissue processing. Using the segmented histology of all trials with tumors (*N* = 17), effective tumor diameters were calculated for the tissue cross-section from the imaging/therapy plane with greater tumor area using the MATLAB function **regionprops()**. Effective tumor diameter was defined as the length of the major axis of the ellipse with the same normalized second central moments as the segmented tumor region.

Previously reported *in vivo* HIFU and bulk US experiments [37] which tested the potential of echo decorrelation imaging to monitor thermal ablation, but did not employ echo decorrelation for automatic feedback control, are referred to here as uncontrolled trials. For consistent comparison with the controlled groups, cumulative echo decorrelation maps for HIFU (*N* = 13) and bulk US (*N* = 10) uncontrolled trials were recomputed using only the first 20 RF frames from each cycle to match the controlled trials reported here.

Successfully controlled trials for both the controlled HIFU and bulk US groups were defined as trials stopped by the control algorithm when Δ_avg_ exceeded Δ_th_. Unsuccessfully controlled trials were defined as trials that were not stopped by the control algorithm, but instead by the predefined maximum number of therapy cycles (*M* = 18). HIFU and bulk US trials were excluded from all statistical analyses if they stopped due to software malfunction or incurred problems in histology processing. Also excluded were HIFU trials with ablation zones that extended from the top to bottom boundaries of the liver lobe, for consistency with exclusion criteria from the previous study reporting the uncontrolled trials [37].

In a *post hoc* analysis, both the controlled and uncontrolled groups were assessed to determine the successfully treated fraction of the control ROI. In this analysis, for HIFU exposures, the control ROI (1 × 1 mm^2^) was placed 2 mm below the tissue surface for both controlled and uncontrolled HIFU trials. For bulk US exposures, the control ROI (18 mm × 6 mm) was placed 2 mm below the tissue surface for both controlled and uncontrolled bulk US trials. The control ROI was considered fully treated if it was completely encompassed by treated tissue (partial or no TTC uptake).

To statistically compare the ablation outcomes of controlled and uncontrolled groups for HIFU and bulk US trials, thermal ablation zone dimensions were characterized for each trial. Width, depth, and area of treated regions (partial or no TTC uptake) were computed from segmented histologic images using a custom MATLAB application. Ablation zone depth was defined as the difference along the array axis between the tissue surface and the deepest treated point. Ablation zone width was defined as the difference between the left and right edges of the treated region along the azimuthal direction at half the measured ablation zone depth. Ablation zone area was calculated as the total area of all pixels classified as treated tissue. Ablation rate was computed as the treated area (cm^2^) per unit treatment time in minutes (min). For bulk US treatments, ablation zone depths were excluded from the analysis because all treated regions extended from the top to bottom boundaries of the rabbit liver lobe.

Statistical analysis of ablation zone dimensions and ablation rate were done using R software (R Foundation, Vienna, Austria). Means and standard errors of ablation zone width, ablation zone area, and ablation rate were computed for the controlled and uncontrolled groups. Normality of data was tested using the Shapiro-Wilk test [50] with the significance criterion *p* < 0.05. Equality of variances between the controlled and uncontrolled groups was tested using the two-sample *F*-test. For normally distributed groups with equal variances, the difference in means was tested statistically using the two-sample *t* test (significance criterion *p* < 0.05). For non-normally distributed groups with equal variances, the difference in medians was tested statistically using the two-sample Wilcoxon signed-rank test [51] (significance criterion *p* < 0.05). For non-normally distributed groups with unequal variances, the difference in cumulative data distributions was tested statistically using the two-sample Kolmogorov-Smirnov (KS) test [52] (significance criterion *p* < 0.05).

Prediction of local US thermal ablation in rabbit liver and VX2 tumor using echo decorrelation imaging was assessed by computing receiver operating characteristic (ROC) curves and area under the ROC curve (AUC) values [36, 37]. ROC curves were computed by comparing thresholded corrected cumulative echo decorrelation images to segmented binary masks for each trial, resulting in parametric plots of the true-positive prediction rate (Sensitivity) vs. false-positive prediction rate (1 − Specificity) [53]. These quantities are respectively defined as Sensitivity = TP/(TP + FN) and 1 − Specificity = FP/(FP + TN), where TP (true positives) is the number of correctly predicted ablated points, FP (false positives) is the number of incorrectly predicted unablated points, TN (true negatives) is the number of correctly predicted unablated points, and FN (false negatives) is the number of incorrectly predicted unablated points. AUC was computed using the trapezoidal rule. ROC curves and AUC values were computed separately for echo decorrelation prediction of treated regions in liver and VX2 tumor for HIFU exposures, bulk US exposures, and all exposures combined within both the controlled and uncontrolled groups.

AUC values were tested for statistical significance against the null hypothesis (AUC = 0.5) using a one-tailed *z* test on the test statistic *z* = (AUC − 0.5)/SE, where SE is the AUC standard error estimated by an established general model [53]. Differences between AUC values (controlled vs. uncontrolled groups and VX2 tumor vs. liver) were tested using the method of DeLong et al. [54, 55] (significance criterion *p* < 0.05, two-tailed). Statistical tests of AUC were adjusted using effective sample sizes determined from the maximum packing density (hexagonal packing) of circular windows with diameter matching the spatial resolution of echo decorrelation images (*d* = 2.35 mm for a Gaussian correlation window width *σ* = 1 mm) as previously described [36, 37].

Differences between average cumulative decorrelation (log_10_-scaled decorrelation per ms) values in treated vs. untreated rabbit liver and VX2 tumor were tested statistically using the two-sample *t*-test (one-tailed, significance criterion *p* < 0.05) for both the controlled and uncontrolled groups. Differences between average cumulative decorrelation values for controlled versus uncontrolled trials, or in VX2 tumor versus rabbit liver, were tested using the two-sample *t*-test (two-tailed, significance criterion *p* < 0.05), as previously employed by Fosnight et al. [37].

## Results

Tissue sectioning results revealed that 22 out 24 implanted VX2 tumors were successfully grown in two weeks. Mean and standard deviation of measured effective diameters for the treated VX2 tumors (*N* = 17) was 9.57 ± 5.24 mm.

Four controlled trials were excluded from further analysis, including 2 trials out of 14 attempts for the controlled HIFU group and 2 trials out of 10 attempts for the controlled bulk US group. One HIFU trial was stopped by the control algorithm after only one therapy cycle when Δ_avg_ exceeded Δ_th_, resulting in a very small HIFU ablation zone and inconclusive TTC-stained histology. Another HIFU trial with an ablation zone extending from the top to bottom boundary of the liver lobe was also excluded, for consistent comparison with previous uncontrolled *in vivo* ablation trials [37]. One bulk US trial was stopped prematurely due to synchronization problems in data acquisition, resulting in erroneous, artifactual high decorrelation. Another bulk US trial encountered a software malfunction due to an unknown operating system error, leading to a sudden restart of the computer performing data acquisition and echo decorrelation imaging, thus prematurely ending that trial.

The modified closed loop control algorithm, employing similar stopping criteria for both HIFU (*N* = 12) and bulk US (*N* = 8) ablation experiments, successfully ceased treatment when the control threshold (log_10_-scaled echo decorrelation per ms: −2.3) was exceeded in all of the remaining 20 controlled trials. Treatment (defined as a region of partial or no TTC uptake) was confirmed in all successfully controlled trials for both series of experiments. For bulk US treatments, the fraction of the control ROI treated was 99.5% ± 1.1% (mean ± standard deviation) for controlled trials and 98.5% ± 5.5% for uncontrolled trials, with 100% of the control ROI treated in 7 of 8 controlled trials and 8 of 10 uncontrolled trials. For HIFU treatments, the treated fraction of the control ROI was 87.9% ± 19.7% for controlled trials and 87.5% ± 29.7% for uncontrolled trials, with 100% of the control ROI treated in 6 of 12 controlled trials and 10 of 13 uncontrolled trials.

**Fig 3:**
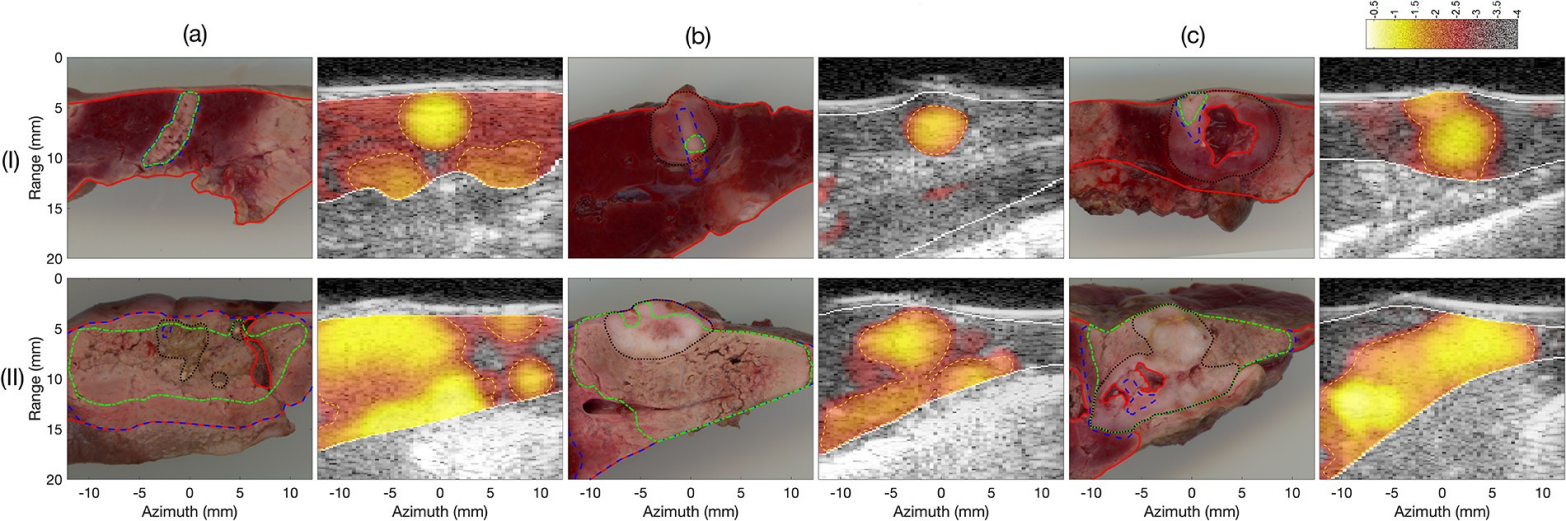
Histologic and hybrid B-mode/echo decorrelation for HIFU and bulk US controlled trials. In the tissue sections, the red, black, blue, and green boundaries indicate the segmented tissue, tumor, treated, and fully ablated regions. In the US images, the white line indicates segmented tissue boundaries and the yellow dashed line represents the optimum prediction threshold for local tissue ablation for all US exposures of both groups.

Histologic and hybrid echo decorrelation/B-mode images for HIFU and bulk US controlled trials are shown in Fig 3. In the histologic images, segmented boundaries are shown for tissue, tumor, and treated (partial or no TTC uptake) regions. In the US images, predicted ablation zone boundaries are outlined based on the optimum prediction threshold for local tissue treatment for bulk US exposures (*N* = 8) using echo decorrelation imaging (log_10_-scaled echo decorrelation per ms: −2.9). Echo decorrelation prediction of thermal ablation shows reasonable agreement with the TTC-stained histology for HIFU and bulk experiments, except for the trial treating a tumor with a large necrotic core, as shown in Fig 3I(C).

**Fig 4:**
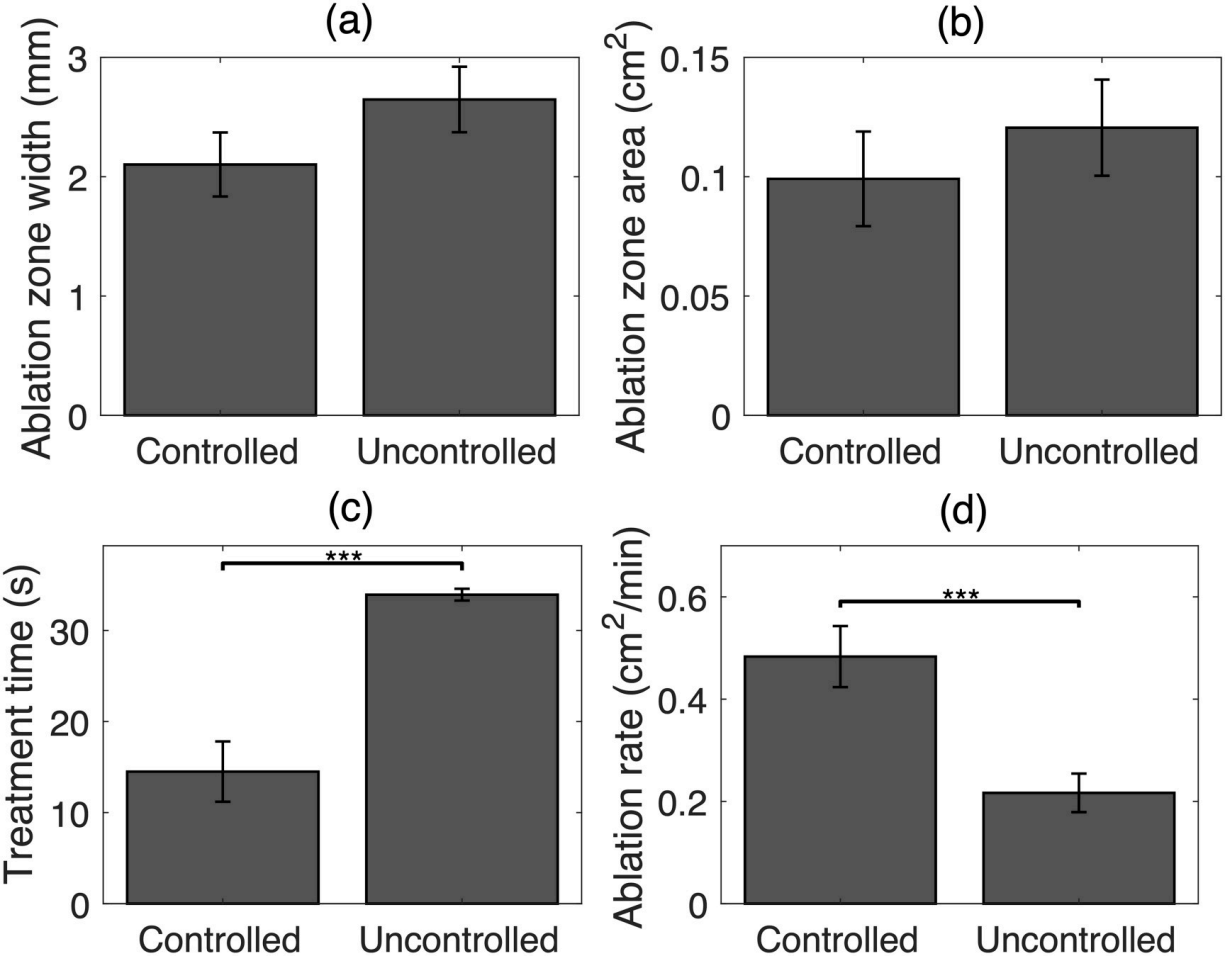
Statistical analysis of ablation results in rabbit liver and VX2 tumor for controlled and uncontrolled HIFU trials. Means and standard errors of (A) ablation zone width, (B) ablation zone area, (C) treatment time, and (D) ablation rate. (*** *p* < 10^−3^).

Statistical analyses of ablation outcomes and treatment time for all successfully controlled (*N* = 12) and uncontrolled (*N* = 13) HIFU trials are shown in Fig 4A–4D. Controlled trials had substantially smaller ablation zone widths and areas than uncontrolled trials (2.10 ± 0.27 mm vs. 2.65 ± 0.27 mm and 0.10 ± 0.02 cm^2^ vs. 0.12 ± 0.02 cm^2^), but these differences were not statistically significant (*p* = 0.170 and *p* = 0.457 in unpaired *t* tests, respectively). Ablation zone depths were similar between the two groups (4.69 ± 0.41 mm vs. 4.56 ± 0.54 mm, *p* = 0.842 in unpaired *t* test). Controlled trials had significantly higher ablation rate than uncontrolled trials (0.48 ± 0.06 cm^2^/min vs. 0.22 ± 0.04 cm^2^/min, *p* = 8.4 ⋅ 10^−4^ in unpaired *t* test) and correspondingly smaller treatment times (14.5 ± 3.31 s vs. 33.9 ± 0.64 s, a significant difference in KS test).

**Fig 5:**
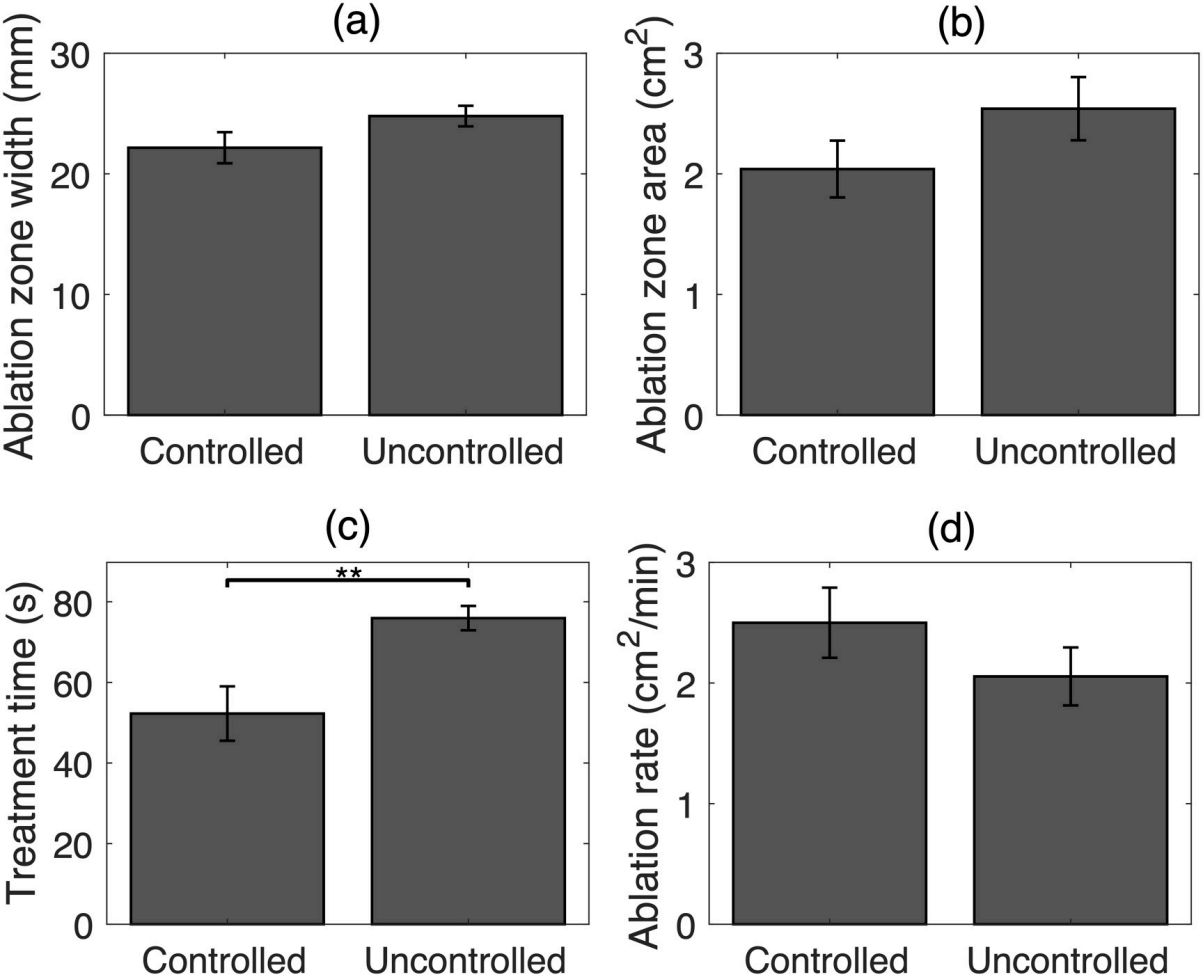
Statistical analysis of ablation results for controlled and uncontrolled bulk US trials. Means and standard errors of (A) ablation zone width, (B) ablation zone area, (C) treatment time, and (D) ablation rate. (** *p* < 10^−2^).

Ablation outcomes and treatment time statistics for controlled and uncontrolled bulk US trials are shown in Fig 5A–5D. Controlled trials resulted in smaller ablation zone widths and areas compared to the uncontrolled group (22.17 ± 1.28 mm vs. 24.79 ± 0.85 mm and 2.04 ± 0.24 cm^2^ vs. 2.54 ± 0.26 cm^2^), but these differences were not statistically significant (*p* = 0.098 in two-sample *t* test for ablation zone width, *p* = 0.351 in Wilcoxon signed-rank test for ablation zone area). Controlled trials were completed in significantly less time than uncontrolled trials (52.28 ± 6.74 s vs. 75.95 ± 3.02 s, *p* = 0.038 in two-sample *t* test) and with a substantially higher ablation rate (2.50 ± 0.29 cm^2^/min vs. 2.05 ± 0.24 cm^2^/min, *p* = 0.251 in two-sample *t* test).

**Fig 6:**
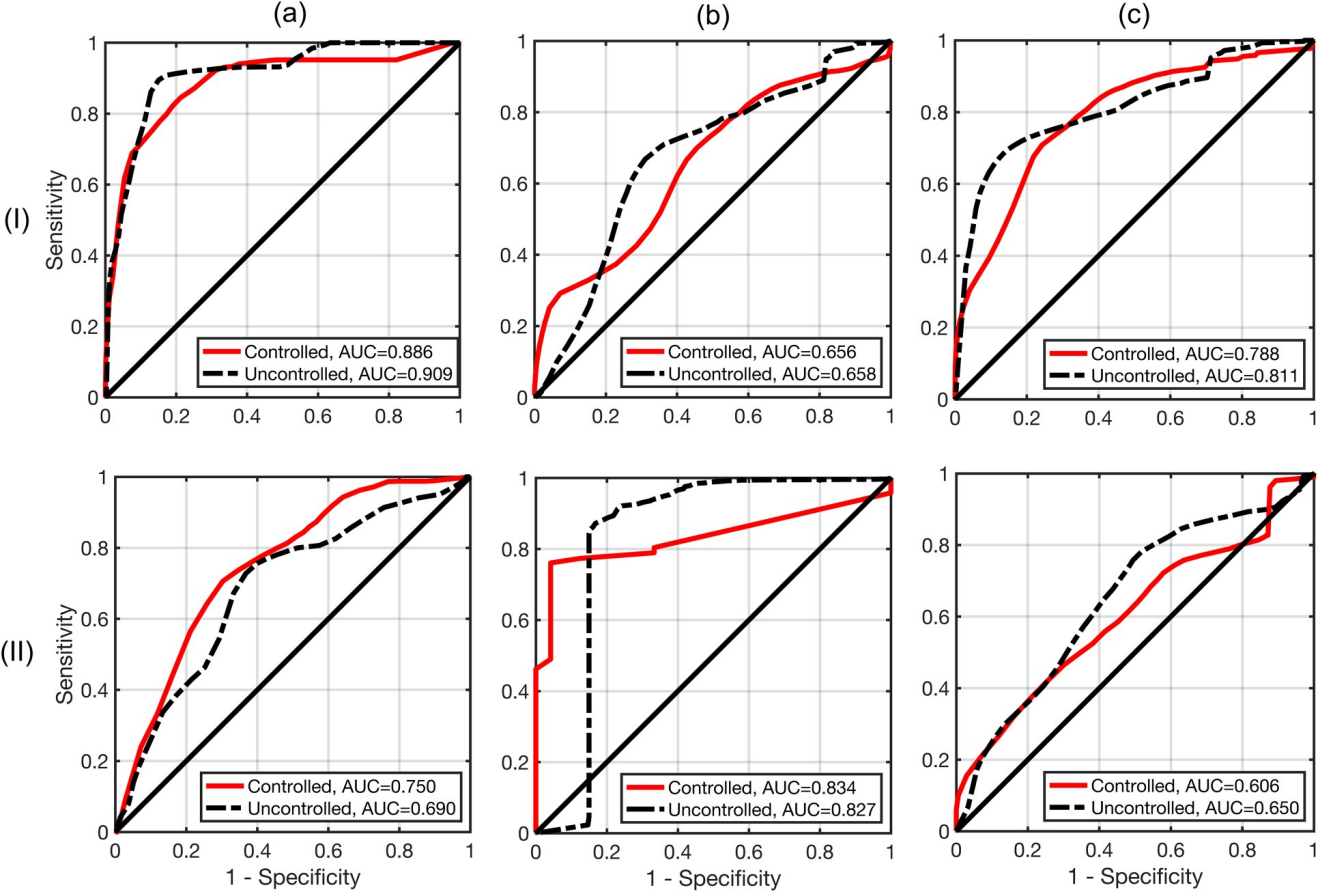
Assessment of prediction capability for echo decorrelation imaging. Receiver operating characteristic curves for echo decorrelation prediction of treatment in rabbit liver (I) and VX2 tumor (II) for HIFU (A), bulk US (B), and all exposures combined (C) for both controlled and uncontrolled HIFU experiments.

ROC curves and AUC values for echo decorrelation prediction of treatment in rabbit liver and VX2 tumor for HIFU, bulk US, and all exposures combined are shown in Fig (6). Statistical analysis results comparing AUC values for the controlled and uncontrolled groups to chance (AUC = 0.5) are shown in Table 1. Echo decorrelation imaging predicted liver and VX2 tumor treatment significantly better than chance in all cases for controlled and uncontrolled trials, with the exception of tumor treatment in controlled bulk US trials, for which prediction assessment was equivocal due to low prevalence of untreated tumor tissue. In all cases, the controlled group showed statistically equivalent prediction capability compared to the uncontrolled group (*p* > 0.1).

**Table 1:** Results of paired, one-tailed *z* tests comparing AUC values for echo decorrelation prediction of treated regions in liver and VX2 tumor vs. chance (AUC = 0.5). Shown for each group is the *z*-statistic expressing the normalized difference between its AUC value and chance, with the corresponding *p* value in parentheses.

	Liver	VX2
HIFU		
Controlled	5.58 (1.19 ⋅ 10^−8^)	2.49 (0.006)
Uncontrolled	8.88 (< 10^−16^)	1.62 (0.052)
Bulk		
Controlled	3.44 (2.9 ⋅ 10^−4^)	1.08 (0.140)
Uncontrolled	3.74 (8.9 ⋅ 10^−5^)	3.39 (3.4 ⋅ 10^−4^)
All exposures		
Controlled	12.43 (< 10^−16^)	1.82 (0.034)
Uncontrolled	19.70 (< 10^−16^)	2.54 (5.6 ⋅ 10^−3^)

**Fig 7:**
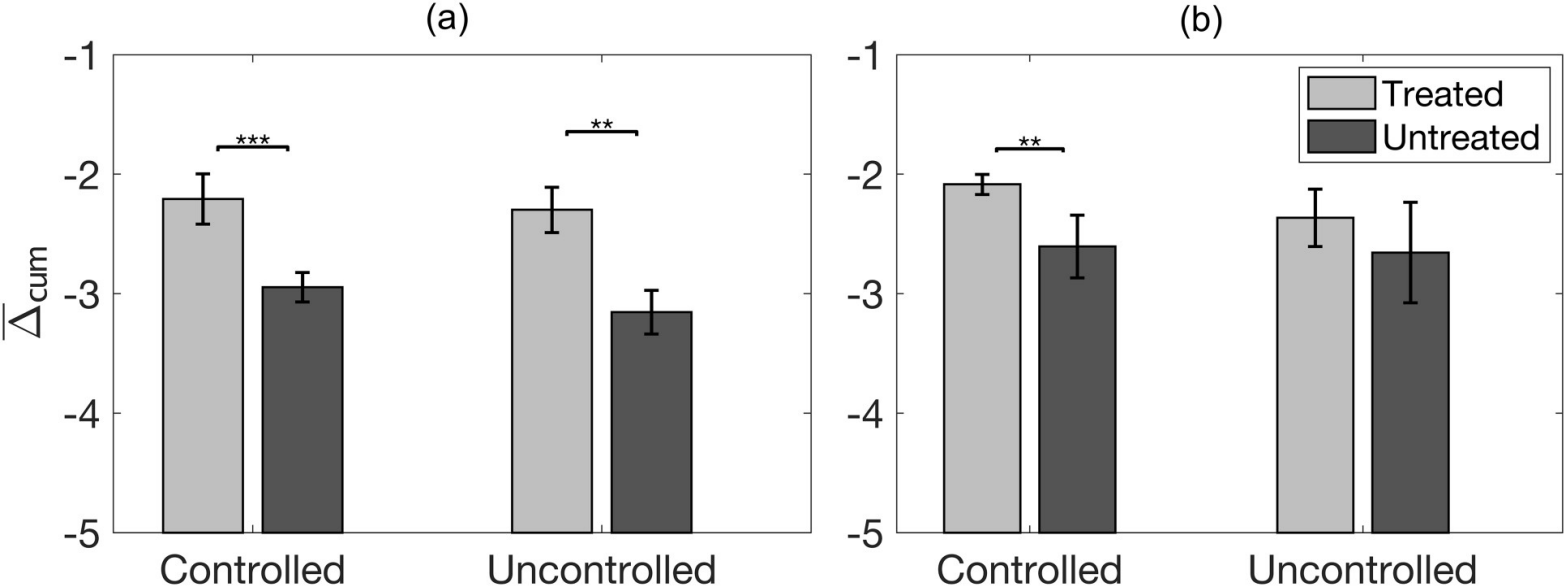
Statistics of cumulative echo decorrelation in treated and untreated tissue. (A) Means and standard errors of log_10_-scaled cumulative decorrelation per ms in treated and untreated rabbit liver for controlled vs. uncontrolled HIFU and bulk US exposures combined. (B) Corresponding statistics for treated and untreated VX2 tumor. (** *p* < 10^−2^ and *** *p* < 10^−3^).

Means and standard errors of the log_10_-scaled cumulative echo decorrelation per ms (Δ_cum_) in treated and untreated rabbit liver and VX2 tumor are shown in Fig 7A and 7B, respectively. For rabbit liver, both controlled and uncontrolled trials had significantly greater mean Δ_cum_ in treated than untreated regions (*p* = 4.8 ⋅ 10^−5^, *p* = 1.8 ⋅ 10^−3^, respectively). For VX2 tumor, controlled trials had significantly greater mean Δ_cum_ in treated than untreated regions (*p* = 8.4 ⋅ 10^−3^). Statistics of average Δ_cum_ comparisons between controlled and uncontrolled groups in treated regions of rabbit liver or VX2 tumor, and also between VX2 tumor and rabbit liver in treated regions of the controlled or uncontrolled groups, showed no significant differences (*p* > 0.8).

## Discussion

In this study, the feasibility of controlling HIFU and bulk US thermal ablation using echo decorrelation imaging feedback was demonstrated in *in vivo* rabbit liver and VX2 tumor. The proposed real-time control algorithm was able to cease all successfully controlled HIFU and bulk US treatments (i.e., all exposures except two bulk US treatments ending early due to software errors) when Δ_avg_ exceeded the predefined Δ_th_ (log_10_-scaled decorrelation per ms: −2.3). Tissue treatment was confirmed by TTC vital staining for all successfully controlled HIFU and bulk US trials. For both HIFU and bulk US ablation, controlled trials showed smaller ablation zone width and area, higher ablation rate, and significantly lower treatment time than uncontrolled trials, with equivalent prediction capability. Possible improvements to echo decorrelation imaging feedback for controlling future preclinical or clinical experiments are discussed below.

Average-decorrelation control criteria (control threshold and ROI shape/size) were effective for controlling the HIFU and bulk US treatments reported here. However, further investigation is required for better control performance in future *in vivo* studies. For example, choice of the echo decorrelation control threshold may explain the less effective prediction of local treatment in VX2 tumor relative to liver, observed here for HIFU exposures and all exposures combined. The threshold (log_10_-scaled decorrelation per ms: −2.3) used here was selected from a similar *in vivo* study reported by Fosnight et al. [37], which computed echo decorrelation maps by ensemble averaging more pulse-echo image frames (114 frames) than used here (20 frames). Retrospective analysis of uncontrolled trials indicated that for ensemble averaging over 20 frames, echo decorrelation thresholds corresponding to 90% specificity for local ablation prediction in liver and VX2 tumor for all exposures combined would be −2.3 and −1.6 (log_10_-scaled decorrelation per ms) respectively. In that case, an appropriate control threshold would be −1.6 (log_10_-scaled decorrelation per ms).

Echo decorrelation prediction of liver and tumor treatment in controlled ablation trials was statistically equivalent to uncontrolled trials for all groups, including HIFU exposures, bulk US exposures, and the combination of all exposures. This result is consistent with *ex vivo* studies showing comparable ablation prediction in controlled trials and long-duration uncontrolled trials [42, 43]. If a higher control threshold were employed, such as −1.6 (log_10_-scaled decorrelation per ms, local ablation prediction could be improved for HIFU and bulk US thermal ablation in both liver and tumor tissue, since this larger threshold would result in higher overall decorrelation values, potentially yielding more definitive confirmation of local ablation. Testing of this conjecture would require an additional series of controlled *in vivo* trials, beyond the scope of the study reported here.

Regarding the control ROI shape and size, for controlled HIFU experiments the use of a small control ROI (1 × 1 mm^2^) at the focal zone helped in mitigating the effect of substantial echo decorrelation artifacts, observed outside the focal zone for some HIFU trials (Fig 3I(A) and 3I(C)), on the control algorithm. For controlled bulk US experiments, the use of a control ROI (18 mm × 6 mm) that approximately matched the shape and size of bulk thermal ablation zones in rabbit liver helped in confirming thermal ablation within 99.5% ± 1.1% of that ROI area in successfully controlled trials. These results are consistent with previous *ex vivo* controlled bulk US ablation experiments employing similar stopping criteria in bovine liver [49].

In the experiments reported here, the motion and noise compensation method previously derived by Hooi et al. [38] was implemented in real time to work simultaneously with echo decorrelation imaging. This correction method improves the capability of echo decorrelation imaging for local ablation prediction in rabbit liver and VX2 tumor [37]. However, the compensation method did not significantly affect the control algorithm performance. That is, without applying the compensation method, treatments would have stopped at the same therapy cycle [45]. This may have occurred because the control ROIs for HIFU and bulk US experiments were accurately located at the regions where large echo decorrelation occurred due to thermal ablation. Another possibility is that the correction method could not compensate some additional artifactual echo decorrelation occurring during therapy cycles (e.g., that due to acoustic radiation force), in which case Δ_avg_ values would be nearly the same for corrected vs. uncorrected cumulative echo decorrelation maps. Fig 3I(C) shows an example of substantial echo decorrelation artifacts, possibly due to the effect of acoustic radiation force on trapped fluid inside the necrotic core of a large VX2 tumor. The compensation method could be modified to account for the effect of acoustic radiation force by including image data from the first one or two therapy cycles within the cumulative sham echo decorrelation map.

Complications due to excessive tissue heating in thermal ablation of liver cancer have been previously reported, including thermal injury to the biliary tree, bowel, and other adjacent organs [56]. Such complications may be related to substantial changes in the progress of thermal ablation for temperatures >100°C due to boiling [57], The control algorithm employed here exploits heat-induced echo decorrelation feedback to stop treatments, likely at temperatures higher than 80°C. However, abrupt jumps in echo decorrelation values between successive therapy cycles may lead to treatment cessation after exceeding the boiling temperature in some cases. Precision of US ablation could be improved by tracking echo decorrelation activity between shorter, consecutive sonication pulses. Using this approach, cumulative decorrelation inside the control ROI could potentially cease treatments before extensive tissue boiling. Alternatively, if attainment of higher ablation temperatures is desired in a given treatment modality, a higher echo decorrelation threshold could be defined for treatment control, thus ensuring that treatments continue until large decorrelation values associated with tissue vaporization are observed.

Future studies in rabbits with implanted VX2 tumors [58] could be performed to assess both the immediate accuracy and longer-term outcomes of US ablation controlled by echo decorrelation imaging feedback, using materials and methods adapted from those reported here. To investigate the spatial accuracy of controlled US ablation, controlled treatment plans could be designed with the goal of ablating an entire tumor and a specified margin of normal tissue. Acute ablation effects could then be assessed using vital staining and statistically compared with the targeted ablation geometry. In a potential survivor study, groups of animals could be treated by controlled and uncontrolled ultrasound ablation in a sterile open surgery or noninvasive procedure. Untreated animals would serve as a control group. Follow-up for all animals could be performed using CT or US monitoring for 2 months or until animal death. Survival analysis for the control and treatment groups would be done using the Kaplan-Meier method [59]. Such a study would provide new information on the effectiveness of controlled US ablation using echo decorrelation imaging feedback in improving long-term survival for a rabbit model of metastatic liver cancer.

## Conclusion

In this paper, HIFU and bulk US thermal ablation were successfully controlled using real-time echo decorrelation imaging feedback, corrected for motion and noise, in *in vivo* rabbit liver and VX2 tumor tissue. Controlled trials showed smaller ablation zone area, significantly less treatment time, and higher ablation rate than uncontrolled trials, with equivalent prediction capability. These results indicate that controlling US thermal ablation using echo decorrelation imaging feedback may reduce treatment time and increase treatment reliability for *in vivo* thermal ablation.
